# The 15th Anniversary of *Materials*—Recent Advances in Materials Chemistry

**DOI:** 10.3390/ma19020329

**Published:** 2026-01-14

**Authors:** Katsuhiko Ariga

**Affiliations:** 1 Research Center for Materials Nanoarchitectonics (MANA), National Institute for Materials Science (NIMS), 1-1 Namiki, Tsukuba 305-0044, Japan; ariga.katsuhiko@nims.go.jp; 2 Graduate School of Frontier Sciences, The University of Tokyo, 5-1-5 Kashiwanoha, Kashiwa 277-8561, Japan

For a period of 15 years from its foundation in 2008, *Materials* has provided its readership with superior content, the production of which has been undertaken by active researchers in the field of material science. Over the course of 15 years, materials chemistry has undergone steady advancement, particularly due to the sophisticated control of nanostructures. This aforementioned progress has been complemented by traditional chemical disciplines, including organic chemistry [1,2,3], inorganic chemistry [4,5,6], polymer chemistry [7,8,9], coordination chemistry [10,11,12], supramolecular chemistry [13,14,15], interfacial chemistry [16,17,18], materials chemistry [19,20,21], and biochemistry [22,23,24]. Additionally, the emergence of new fields such as nanotechnology [25,26,27], nanoarchitectonics [28,29,30], and AI-related technology [31,32,33] has significantly contributed to the development of functional materials. These advances have demonstrated efficacy in addressing a range of social problems related to energy [34,35,36], environmental [37,38,39], and biomedical issues [40,41,42]. Material nanoarchitectonics in particular provide opportunities to control material structures, ranging from atom-level arrangements [43] to complex biomaterial-based structures [44]. The journal, *Materials*, has collected research papers from a broad spectrum of material chemistry topics worldwide for publication in this Special Issue titled “Recent Advances in Materials Chemistry”. The papers are summarized and organized throughout this editorial article.

Of course, various kinds of approaches to create new functional materials and attractive material structures have been reported. In addition, theory, analyses, and structure/property investigations on materials have important roles in related fields. Raman classification and pXRF are two examples. Shi et al. published a study on lithium deposition. In this study, the authors utilized electrochemical atomic force microscopy to observe the dynamic evolution of lithium deposition using an in situ electrochemical atomic force microscope and an electrochemical workstation in both etheryl-based and ethylene carbonate-based electrolytes. In addition, application-oriented approaches with functional materials have been actively purused. Applications for devices and the related functional mechanisms are attractive targets. Park et al. developed innovative bipolar host materials based on indolocarbazole for red phosphorescent OLEDs. New indolocarbazole-triazine derivatives were designed and synthesized. The synthesized materials were used to fabricate and test both hole-only and electron-only devices in terms of their carrier mobility.

Demands for energy applications are crucial in current society. Much effort has been made for the production and fabrication of high-efficiency materials. Ji et al. present a review article on the use of doping engineering in manganese oxides for aqueous zinc-ion batteries [45]. Recent developments in manganese-oxide-based cathodes doped for aqueous zinc-ion batteries have been comprehensively reviewed. The contents of this review are as follows: (i) A classification of manganese-oxide-based cathodes is provided; (ii) an examination of the energy storage mechanisms of manganese-oxide-based cathodes; (iii) a discussion on the synthesis route and role of doping engineering in manganese-oxide-based cathodes; and (iv) an analysis of the performance of doped manganese-oxide-based cathodes in aqueous zinc-ion batteries. Chen developed composites of Na_4_Fe_3_(PO_4_)_2_P_2_O_7_ doped with Mo for use in high-rate and long-life sodium-ion batteries. A novel and uncomplicated approach is introduced in this research to improve the electrochemical properties of the cathode materials designed for sodium-ion batteries. Macías et al. created SrTiO_3_-SrVO_3_ ceramics for use as anodes in solid-oxide fuel cells. This study examines methods for manufacturing strontium titanate–vanadate electrodes from oxidized starting materials. It is anticipated that further enhancement of the composite’s electrical performance can be achieved through the optimization of the processing route and microstructure. This optimization will facilitate a more efficient reduction of the oxidized precursor and ensure optimal percolation of the SrVO_3_ phase. Bamburov et al. investigated the effect of processing and thermochemical treatment conditions on the electrical conductivity of SrTiO_3_-derived ceramics with moderate acceptor-type substitution in a strontium sublattice.

Material solutions for environmental and biomedical issues have also received much attention. Wang et al. published a review article on computational materials design for ceramic nuclear waste forms. This methodology employs machine learning, first-principles calculations, and kinetic rate theory. This approach could facilitate accelerated development of ceramic waste forms and improve predictions of their performance for difficult nuclear waste elements. The United Kingdom’s adoption of pyroprocessing for spent nuclear fuel as an alternative to current aqueous processing methods necessitates a robust scientific foundation for all pertinent processes [46].

From these selected examples, it is clear that materials selection, organization and fabrication of materials, and their applications have a huge variety. Continued advancement in these research areas has the potential to yield a vast array of functional materials. This could serve as a transformative approach within the broader field of materials chemistry [47]. The demand for materials could be met using these research developments. Consequently, the advancement of material chemistry has the potential to contribute to the preservation of our planet.
